# Mechanical and Microstructural Comparison of Improved Sand with Guar Gum and Cement

**DOI:** 10.3390/polym18101191

**Published:** 2026-05-13

**Authors:** Jair de Jesús Arrieta Baldovino, Luis Carlos Suárez López, Jesús Alberto Alcalá Vergara, Yamid E. Nunez de la Rosa

**Affiliations:** 1 Civil Engineering Program, Universidad de Cartagena, Cartagena de Indias 130015, Colombia; lsuarezl@unicartagena.edu.co (L.C.S.L.); jalcalav@unicartagena.edu.co (J.A.A.V.); 2 Faculty of Engineering and Basic Sciences, Fundación Universitaria Los Libertadores, Bogota 111221, Colombia

**Keywords:** guar gum, Portland cement, microstructure, soil stabilization, unconfined compressive strength

## Abstract

Research on sustainable alternatives to conventional soil stabilization has promoted the use of natural biopolymers as partial substitutes for cementitious binders. This study presents a mechanical and microstructural comparison between poorly graded Colombian sand stabilized with guar gum (GG) and Type III Portland cement. GG was incorporated at 0.25–1.00% with curing periods of 28 and 90 days, while cement contents ranged from 3 to 9% with 7 days of curing. A total of 108 cylindrical specimens were tested using unconfined compressive strength (UCS) and SEM–EDS analyses. Results show that both binders significantly improve soil strength, although cement exhibits a steeper strength gain due to hydration processes. GG-treated samples reached a maximum UCS of 470 kPa at 90 days, representing an increase of approximately 40% compared to 28 days and showing comparable performance to 5% cement. The porosity/binder index (η/B_iv_) demonstrated a strong correlation with UCS (R^2^ > 0.91), confirming its predictive capability. Microstructural analysis revealed the formation of C–S–H and ettringite in cement-treated samples, while GG-treated soils exhibited hydrogel bridges and the presence of pores that may influence particle bonding. Overall, the results demonstrate the technical feasibility of GG as a sustainable soil stabilization alternative.

## 1. Introduction

The study of environmentally friendly biological alternatives for soil stabilization is a technique widely used in geotechnical engineering that aims to replace traditional or conventional stabilizing binders such as cement and lime [1]. Other cementitious binders frequently employed to improve soil stability include bituminous materials, fly ash, and sodium sulfate [2,3,4,5]. Among these alternatives, biopolymers have emerged as promising materials, as they are considered sustainable and carbon-neutral. They are classified as renewable materials since they are produced from non-food agricultural crops and offer several advantages in terms of abundance, non-toxicity, and their potential to reduce greenhouse gas emissions [6,7].

The behavior of cemented soils exhibits characteristics that differ from those of conventional soils in geotechnical engineering. Properties such as stiffness and deformability are influenced by the presence of a cementing agent. Consequently, cemented soils can sustain higher stress states than those observed in uncemented soils when both materials are compared under the same porosity conditions. The use of artificial techniques for soil cementation is generally directed toward two main applications. In laboratory conditions, the addition of cementing agents to soils is primarily aimed at stimulating the behavior of naturally cemented soils. This approach allows for reduced costs and minimizes the difficulties associated with obtaining high-quality undisturbed samples. On the other hand, the use of cementing agents in field applications aims to produce geomaterials that are geotechnically compatible with the strength and deformability demands required at a given site.

Sustainable biopolymers offer benefits that go beyond the environmental advantages of conventional stabilizers commonly used in construction. Soils treated with biopolymers exhibit a significant increase in shear strength and a reduction in the shrinkage behavior of expansive materials [8]. Among the most commonly used biopolymers are gellan gum, Xanthan Gum (XG), Guar Gum (GG), and Agar–Agar Gum (AG-A). These biopolymers act by forming gel-like structures that bind soil particles together, thereby enhancing cohesion and overall stability [1,6,9,10]. Biopolymers are also employed for the stabilization of surface soils due to the improvement in effective cohesion they provide, which results in increased resistance against shallow slope failures [11].

GG is a high-molecular-weight natural polysaccharide derived from the endosperm of guar seeds (*Cyamopsis tetragonoloba*). It is more economical compared to other biopolymers such as xanthan gum and gellan gum. In addition, GG dissolves readily in cold water, increasing the viscosity of the aqueous system and acting as an efficient non-ionic stabilizer and hydrocolloid [12,13]. It has been demonstrated that a 2% GG inclusion produces the most significant modification of soil geotechnical properties, representing an attractive improvement achieved through ecological and durable means when compared to alternatives such as cement [6,14]. Furthermore, GG significantly reduces permeability across different soil densities by promoting particle adhesion and pore filling [15].

In the case of a sand–bentonite mixture stabilized with GG, lower strength was observed compared to samples stabilized with xanthan gum, along with a higher degree of time-dependent deformation and evidence of biological degradation after 180 days. These results suggest that GG may not be suitable as a long-term stabilizer unless it is combined with antimicrobial agents [16]. Conversely, no evidence has been found indicating that GG negatively affects plant germination, suggesting that it provides a favorable environment for vegetation growth [17]. Other studies have reported that GG has served as an alternative agent for soil stabilization, improving soil properties through the formation of gels and biofilms that encapsulate soil pores and promote uniform matrix distribution via hydrogel formation during hydration. Additionally, GG has been used as a liner material in landfills, serving as a replacement for traditional bentonite [18,19,20,21].

For brittle cementing agents, the cementation bonds tend to break at very small strains, after which the frictional component is mobilized at larger deformations. The effects of cementation on strength and deformation parameters indicate that, in sands, cementation introduces an apparent cohesive intercept and tensile strength. An important phenomenon is the difference in volumetric expansion and the strain level at which it occurs. Experimental evidence suggests that the mobilization of strength in cemented soils differs from that in uncemented soils, not only due to the presence of bonding but also due to additional volumetric strain energy components that influence the stress–strain response of soil–cement mixtures.

Several factors affect this behavior, including the amount of added water, which reflects the degree of cement hydration, the curing time, and the binder content. In general, peak strength increases with curing time as the bonding structure develops. At high confining stresses, however, the effect of cementation on shear strength is significantly reduced due to the shearing and breakage of larger particles formed by the bonding of smaller grains. As a result, cemented soils do not exhibit markedly different dilation rates compared to compacted uncemented soils under such conditions. Additionally, the stress paths of cemented soils tend to show pronounced curvature, reflecting the progressive degradation of bonding during loading.

Previous studies have highlighted the potential of biopolymers as sustainable alternatives for soil stabilization, demonstrating improvements in shear strength and reductions in shrinkage behavior. These materials, including guar gum (GG), xanthan gum, and gellan gum, act by forming gel-like structures that bind soil particles and enhance overall cohesion. Additionally, biopolymer treatment has been associated with reduced permeability due to pore filling and improved particle interaction, contributing to enhanced soil stability and durability [22].

In the present study, sand obtained from an open-pit mine located in the municipality of Luruaco (Atlántico), Colombia, was used. This sand was classified as poorly graded (SP) according to the Unified Soil Classification System (USCS). This soil type was previously investigated in a study by Lopez et al. [23], where it was cemented using Type III Portland cement as a binding agent to produce soil–cement cylinders at dosages of 3%, 5%, 7%, and 9%, cured for 7 days. The specimens were subjected to UCS tests and SEM–EDS analysis. In the present work, the same sand is stabilized using GG with dosages of 0.25%, 0.50%, 0.75%, and 1.00%, and curing periods of 28 and 90 days. The resulting specimens are subjected to the same tests conducted in the previous study. The main objective is to compare the results obtained using GG with those derived from the same soil stabilized with Type III Portland cement in order to evaluate the geotechnical improvements provided by a traditional cementitious binder relative to a biopolymer such as GG. This comparison aims to contribute to a future perspective in which biopolymers may be used as cement replacements, thereby helping to reduce the carbon footprint associated with cement production. Unlike studies focused solely on strength enhancement, this research aims to provide a combined mechanical and microstructural understanding of GG-stabilized soils, contributing to the identification of underlying stabilization mechanisms.

## 2. Materials and Methods

### 2.1. Materials

#### 2.1.1. Sand

The soil used in this study is a poorly graded sand (SP) previously characterized by López et al. [23] and Vergara et al. [24]. It originates from the municipality of Luruaco, Atlántico, Colombia, located at 10°37′38.34″ N and 75°13′0.17″ W. The material is extracted from an open-pit operation and subsequently subjected to crushing and washing processes.

**Figure 1:**
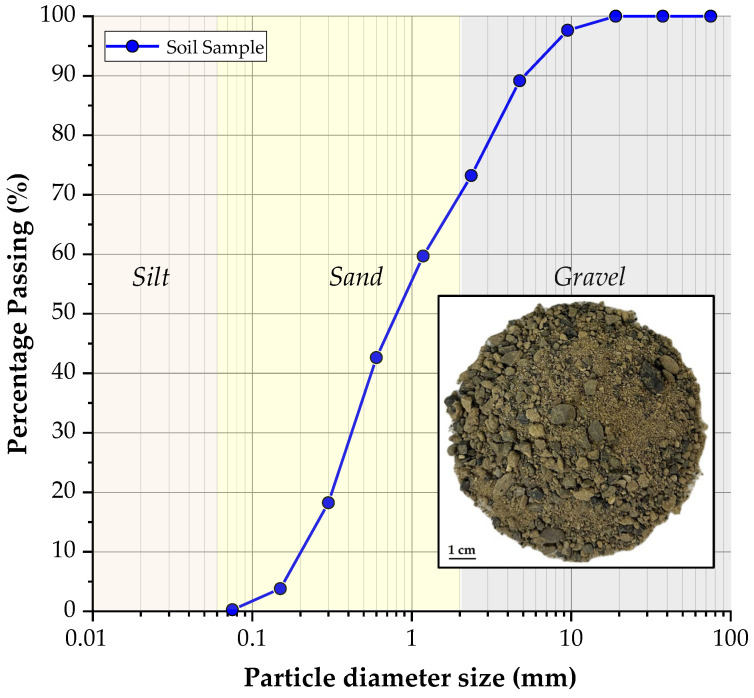
The granulometric curve of the soil sample.

Figure 1 shows the particle-size distribution curve of the sand, while Table 1 summarizes its geotechnical properties. According to the Unified Soil Classification System USCS [25], it is classified as poorly graded sand (SP). Its specific gravity (Gs = 2.73) was determined by the pycnometer method in accordance with ASTM D854 [26]. Figure 1 also shows a photo of the sand sample. In terms of granulometric analysis, the soil is composed of 10.8% gravel, 20.1% coarse sand, 42.3% medium sand, 26.6% fine sand, and 0.2% silt. The mean diameter of 50% passing (*d*_50_) is 0.85 mm, and the effective diameter is 0.20 mm. In addition, the coefficient of curvature was calculated at 0.7, and the uniformity coefficient was 5.9.

**Figure 2:**
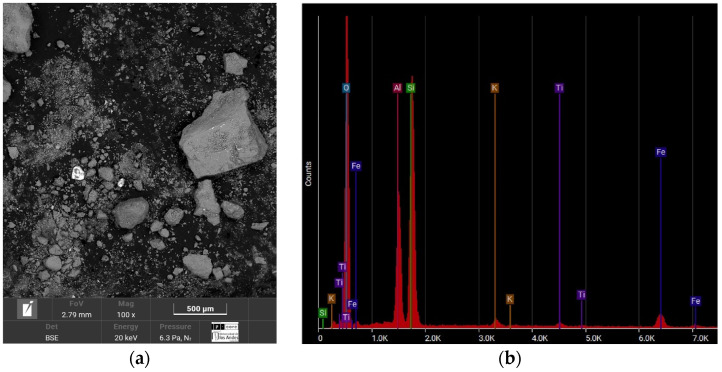
SEM-EDS test result of the Luruaco sands. (**a**) Microstructure and (**b**) elemental composition.

According to SEM–EDS analyses (Figure 2), the sample shows the presence of oxygen (O), silicon (Si), and aluminum (Al), which are typical elements of natural quartzitic sands. The presence of iron (Fe) is also observed, commonly found in soils from the Colombian Caribbean region due to weathering processes [23].

#### 2.1.2. Guar Gum

**Figure 3:**
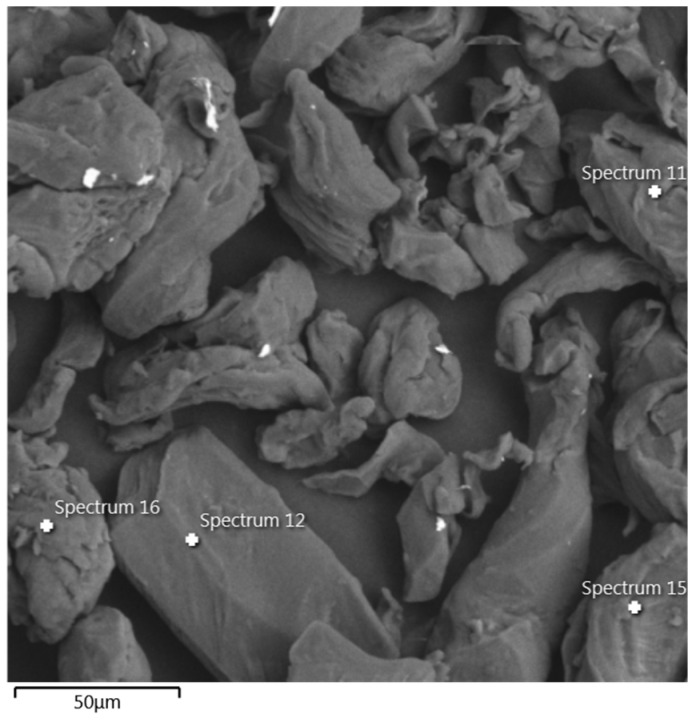
GG morphology recorded in the SEM analysis.

The GG employed in this study (Figure 3) is a natural polysaccharide obtained from the endosperm of *Cyamopsis tetragonoloba*. Its chemical structure consists of a linear backbone of β-(1 → 4)-D-mannose with galactose side chains linked through α-(1 → 6) bonds, resulting in a mannose-to-galactose ratio typically between 1:5 and 2:1 [29,30,31,32]. The GG used in this study has a density of 0.92 g/cm^3^ and, according to Flores et al. [33], dissolves readily in water, developing high viscosity that decreases with increasing shear rate, reflecting its pseudoplastic behavior.

### 2.2. Methodology

#### 2.2.1. Specimen Molding and Preparation

**Figure 4:**
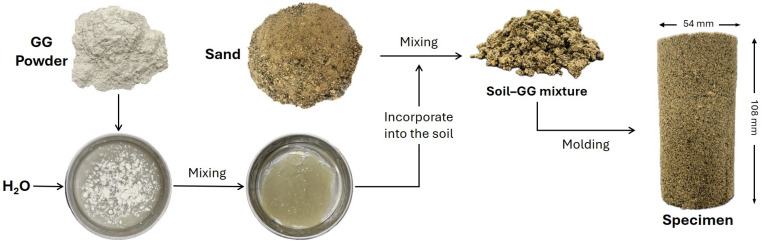
Flowchart of the manufacturing process of GG-stabilized specimens.

Distilled water was used in the preparation of the specimens and was combined with the different dosages, as indicated in Table 2. These proportions were selected based on ranges commonly reported in the literature for GG [34,35,36,37]. The GG content, together with the water content (10%), which was defined with respect to the dry unit weight of the soil, was established to ensure suitable mixing conditions. The blend was prepared manually, incorporating it into the soil until a uniform mixture was achieved, as illustrated in Figure 4. The three molding densities of the specimens were selected within the range defined by the minimum and maximum densities of the sand. A total of 72 GG–soil specimens were prepared.

As the specimens are cemented, after compaction of the final layer and weighing of the mold–specimen assembly, the set is sealed in plastic bags and stored for 48 h to allow the cement to set and minimize moisture loss during curing. After this period, the specimen is extruded from the mold and stored in sealed plastic bags until it reaches the designated testing age.

A total of 36 specimens were prepared using conventional sand–cement mixtures, with Type III Portland cement employed as the binding agent. Cement contents ranging from 3% to 9% were adopted, as presented in Table 2. The compaction densities were kept consistent with those used for the GG–soil mixtures.

All 108 specimens were molded in cylindrical form, maintaining a 1:2 aspect ratio, corresponding to 108 mm in height and 54 mm in diameter, in accordance with ASTM D1631 [38]. The specimens were prepared in three layers, ensuring proper bonding and uniformity between them, thereby achieving consistent physical characteristics across all samples.

The specimens were subjected to the acceptance criteria reported in the literature [39,40,41]. The GG mixtures were cured for 28 and 90 days, whereas the cement-treated specimens were cured for 7 days.

The different curing periods were selected considering the distinct hardening mechanisms of both binders. Type III Portland cement was evaluated at 7 days because it attains a high percentage of its compressive strength at early ages due to its rapid hydration process. In contrast, GG-treated sand was evaluated at 28 and 90 days to capture the progressive development of polymeric bonding and hydrogel formation, which may require longer curing periods to produce measurable improvements in strength and microstructure.

#### 2.2.2. UCS Test

Subsequently, unconfined compression strength (UCS) tests were performed following the procedures outlined in ASTM D2166 [42]. During the test, the samples were subjected to an axial load by means of a multi-test hydraulic press with a capacity of 50 kN and a sensitivity of 0.1 kN, which operated at a constant deformation rate of 1.15 mm/min.

#### 2.2.3. Application of the Porosity–Binder Index in the Prediction of Unconfined Compressive Strength

The porosity/binder index ($\eta /B_{iv}$), originally proposed by Consoli et al. [43] and later validated by Baldovino et al. [44], has proven to be an effective parameter for predicting the mechanical behavior of soils stabilized with different types of binders. In this study, the same approach is applied to the Sand–GG and Sand–Cement mixtures to evaluate their unconfined compressive strength (UCS).

The volumetric binder content ($B_{iv}$) was determined using the formulation proposed by Baldovino et al. [39], while the porosity (η) was adapted to incorporate the effects of GG addition, as shown in Equation (1):

(1) $$\eta =100-\frac{100\cdot \gamma_{d}\cdot V_{s}}{V_{specimen}}\cdot \left[\frac{1}{G_{s}\cdot \left(1+\frac{{GG}_{\%}}{100}\right)}+\frac{{GG}_{\%}}{100\cdot \rho GG\cdot \left(1+\frac{{GG}_{\%}}{100}\right)}\right]$$

Equation (1) incorporates the GG content $\left({GG}_{\%}\right)$, the material densities described in Section 2.1.2, the specific gravity of the soil ($G_{s}$) listed in Table 1, the molding densities ($\gamma_{d}$) reported in Table 2, along with the specimen volume ($V_{specimen}$) and the soil volume ($V_{s}$).

The UCS ($q_{u}$) are related to the η/B_iv_ index by means of adjustments based on two empirical exponents (*a* and C) and an empirical constant A_q_ [39]. These properties are expressed in kPa, as defined in Equation (2):

(2) $$q_{u}=A_{q}{\left[\frac{\eta }{{\left(B_{iv}\right)}^{a}}\right]}^{-C}$$

#### 2.2.4. Statistical Analysis of Factors Influencing the UCS of GG-Soil Samples

The statistical analysis involved an analysis of variance (ANOVA) to assess the factors affecting the UCS of stabilized GG-soil specimens. Both the individual effects and their interactions were considered, including density, GG content, curing time, density × GG, density × curing time, GG × curing time, and density × GG × curing time. The analysis was performed using IBM SPSS Statistics 31, adopting a 5% significance level (*p* < 0.05), to identify statistically significant differences among the evaluated treatments.

#### 2.2.5. Microstructural Analysis

Scanning electron microscopy (SEM) analyses were performed on strategically selected areas of different specimens, reaching magnifications of up to 5000×. These observations were performed on both the sand–GG and sand–cement samples to identify and compare their microstructural differences. The analyses were carried out using a LYRA-3 dual-beam SEM–FIB system manufactured by TESCAN (Tescan Orsay Holding, Brno–Kohoutovice, Czech Republic).

## 3. Results and Discussion

### 3.1. Influence of Cement and GG Content, Dry Unit Weight, and Curing Time on the Strength of a Stabilized Sandy Soil

**Figure 5:**
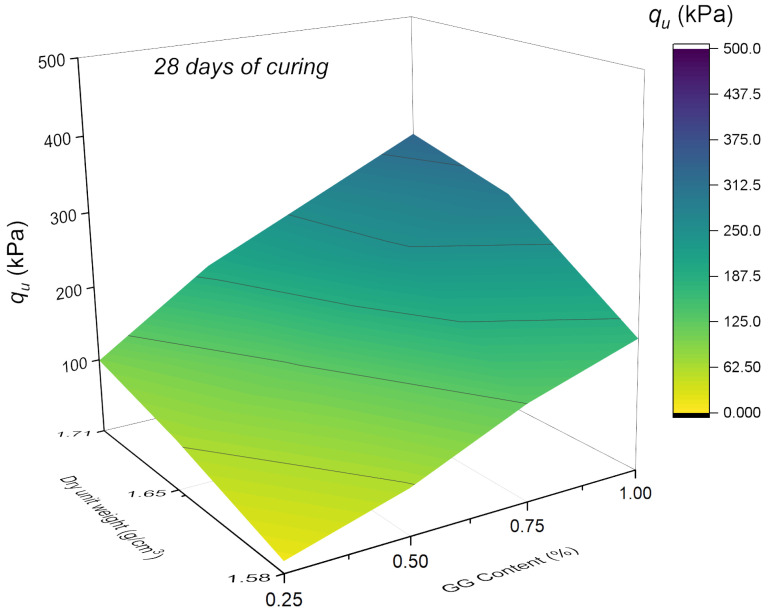
Effect of GG content (%) and dry unit weight (g/cm^3^) on the UCS of GG–soil cured for 28 days.

**Figure 6:**
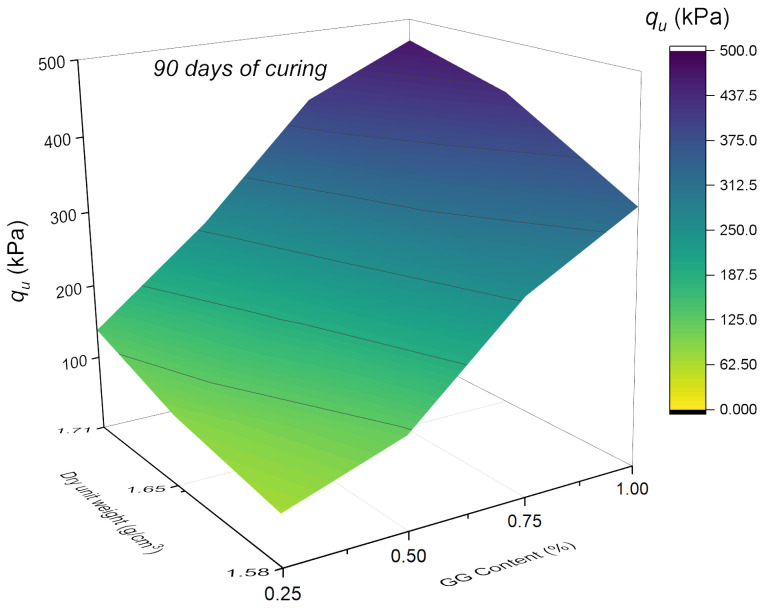
Effect of GG content (%) and dry unit weight (g/cm^3^) on the UCS of GG–soil cured for 90 days.

In Figure 5 and Figure 6, the data are plotted in 3D maps that illustrate the soil stabilization with GG at 28 and 90 days, respectively. According to the UCS results, both GG content and curing time have a positive influence on mechanical behavior. For instance, at 28 days and a dry unit weight of 17.1 g/cm^3^, the average strength increases from 99.17 kPa at 0.25% to 199.18 kPa at 0.5%. This upward trend continues at 0.75%, reaching 266.62 kPa, and further rises to 334.33 kPa at 1% GG, showing a consistent increase with higher biopolymer content.

At 28 days, a maximum strength of 334.33 kPa was obtained for 1% GG, whereas at 90 days, under the same density and GG content, the strength reached 470.40 kPa, representing a 40.7% increase compared to 28 days. A similar comparison across curing times shows that for 0.75% GG, the increase is 52.3%, confirming the significant influence of curing time on UCS.

These results are consistent with those reported by Sujatha and Saisre [45], who observed that the strength of soil treated with 2% GG increased from 221.7 kPa without curing to 463.6 kPa at 90 days, corresponding to an increase of nearly 52%. The authors attributed this behavior to the formation of hydrogen bonds among the numerous hydroxyl groups present in the chemical structure of GG and to the action of hydrocolloids generated through these interactions, which enhance the cohesion of the soil–biopolymer system. Overall, both studies demonstrate that GG content and curing time are key variables governing the progressive increase in mechanical strength.

**Figure 7:**
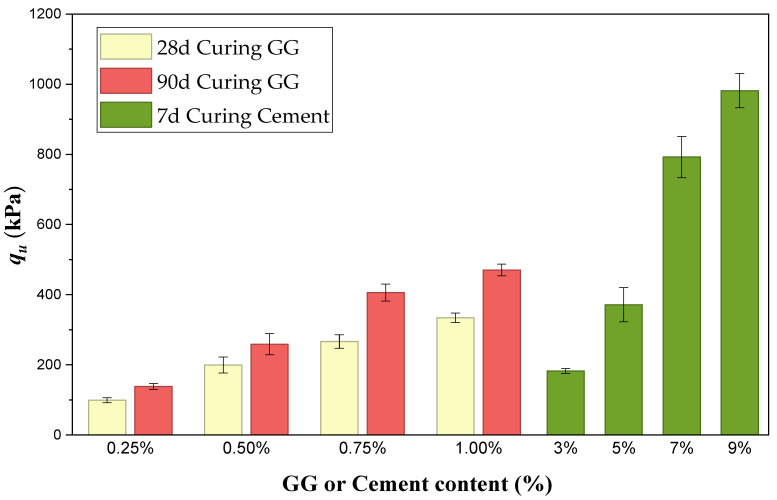
Comparison of UCS of GG-soil and cement–soil at different curing periods (dry unit weight = 1.71 g/cm^3^).

Figure 7 shows the UCS behavior of the stabilized soils considering the highest molding dry density (1.71 g/cm^3^). It is evident that GG-stabilized mixtures increase in strength when cured from 28 to 90 days, particularly at higher GG contents. Likewise, cement achieves higher strength values starting from 7% cement content, reaching values close to 1000 kPa. However, when comparing the best GG scenario—1% GG content and 90 days of curing—this strength is approximately 27% higher than that of the 5% cement mixture and represents an improvement of more than 158% compared to the 3% cement mixture.

It is important to note, however, that the difference in strength development over time is significant, as the cement reaches these high values in just 7 days of curing. Therefore, in scenarios where high and rapid UCS is required, cement remains the superior option due to its dense and chemically stable cementitious matrix. For moderate applications with longer curing times, GG could be considered a technically viable alternative.

In a similar study, Zhang et al. [46] found that guar gum (GG) enhances cohesion between soil particles through the formation of a hydrated gel, creating a synergistic “bond–bridge” system that improves the mechanical properties of the soil. On the other hand, certain damaged bonds between the soil and the biopolymer can be restored through appropriate treatment; specifically, wetting–drying cycles may reactivate the binding properties of biopolymers, thereby modifying previously broken bonds within the soil matrix [47].

### 3.2. Use of the Porosity/Binder Index to Predict the Strength

**Figure 8:**
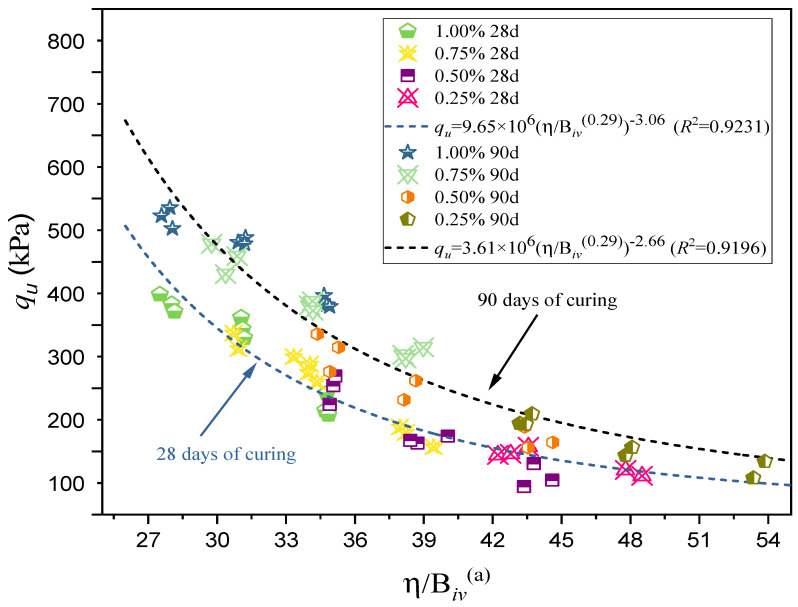
Porosity/binder index vs. UCS of sand–GG mixtures at 28 and 90 days of curing.

**Figure 9:**
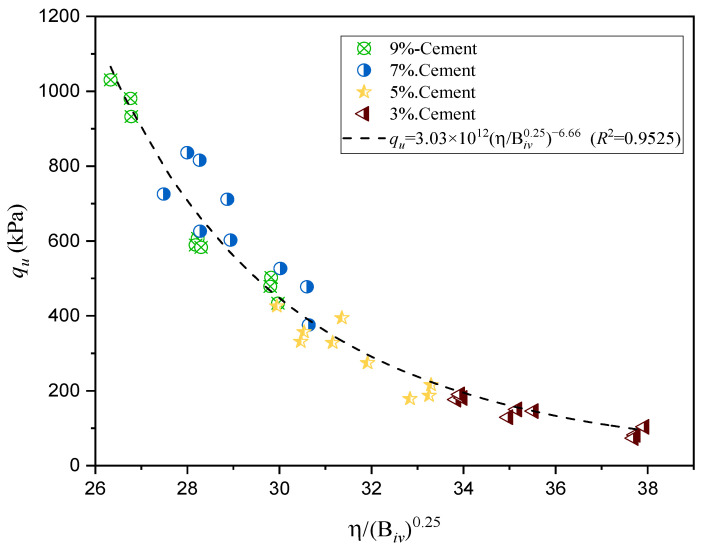
Porosity/cement index vs. UCS for soil–cement mixtures cured for 7 days.

The applicability of the porosity/binder index $\left(\eta /B_{iv}^{a}\right)$ for predicting UCS is illustrated in Figure 8 and Figure 9.

As shown in Figure 8, sand stabilized with GG at 28 and 90 days exhibits a clear inverse power-law relationship between the index $\eta /B_{iv}^{0.29}$ and UCS. These curves are presented in Equation (3) for 28 days of curing and Equation (4) for 90 days of curing.

Gelation of GG occurs when the polymer hydrates and its molecular chains expand in the presence of water, forming a viscous hydrogel capable of coating sand particles and creating polymeric bridges between them. These bridges contribute to particle bonding and partial pore filling, improving the mechanical response of the treated sand. However, the efficiency of this mechanism depends on the available moisture, GG content, curing time, dry density, particle arrangement, and mixture homogeneity.

(3) $$q_{u}=9.65\times 10^{6}{\left[\frac{\eta }{{\left(B_{iv}\right)}^{0.29}}\right]}^{-3.06}\left(R^{2}=0.9231\right)$$

(4) $$q_{u}=3.61\times 10^{6}{\left[\frac{\eta }{{\left(B_{iv}\right)}^{0.29}}\right]}^{-2.66}\left(R^{2}=0.9196\right)$$

Figure 8 confirms that, as the porosity/binder index decreases, the compressive strength increases. Reliability fits greater than or equal to 0.91 for both curing periods indicate that the index successfully unifies mixtures with different GG contents (0.25%, 0.50%, 0.75%, and 1.00%) and curing times within this predictive approach.

Baldovino et al. [44] reported that, for XG–clay mixtures, the exponent “*a*” is 0.04. Their results suggest that in fine-grained cohesive soils treated with biopolymers, porosity has a stronger influence on behavior than the binder content. In contrast, the higher value obtained in this study for GG-treated sand (0.29) indicates that, in granular soils, the role of the binder becomes more significant. This can be explained by the fact that sand lacks inherent cohesion and therefore depends on the binder to create links between particles and improve its mechanical response. In this sense, incorporating a fine fraction could help enhance the matrix, which has already been observed for XG by Abbasi et al. [48], who used an 80–20% sand–clay mixture to achieve better mechanical performance after stabilization with XG.

The porosity/binder index is affected by both the void structure of the compacted sand and the volumetric amount of GG available to generate bonding. Lower porosity and higher effective GG content reduce the η/B_iv_ ratio, favoring stronger interparticle connections and higher UCS values. Conversely, excessive porosity may interrupt the continuity of the hydrogel matrix and limit strength gain.

On the other hand, Figure 9 presents the UCS behavior of sand–cement mixtures cured for 7 days. The regression model is reported in Equation (5):

(5) $$q_{u}=3.03\times 10^{12}{\left[\frac{\eta }{{\left(B_{iv}\right)}^{0.25}}\right]}^{-6.66}\left(R^{2}=0.9525\right)$$

The coefficients of determination (R^2^) for the GG–soil mixtures (0.9231 and 0.9196) at 28 and 90 days, respectively, indicate that the porosity/binder index not only shows high reliability but also strong predictive capability for UCS in alternative systems such as GG-treated soils. This is consistent with the findings of Consoli et al. [43], who stated that scalar A reflects the combined influence of bonding strength and the frictional resistance of the soil matrix. Furthermore, the observations by Diambra et al. [49] and Festugato et al. [50] regarding the dependence of the index exponents on soil type and bonding mechanisms agree with the trends identified in the present analysis.

Soils and soil–cement mixtures consist of solid particles with voids filled by air and water. Field compaction is not sufficient to eliminate all air voids; therefore, soil–cement should be considered as inherently incompletely compacted. The strength of incompletely compacted materials cannot be directly related to the water–cement ratio, since such relationships are only valid for fully compacted systems where air voids have been effectively eliminated. Instead, previous studies have demonstrated that the strength of partially compacted materials is more appropriately related to the void–cement ratio. This concept provides a more realistic framework for describing mechanical behavior under typical geotechnical conditions. In this context, the porosity/binder index (η/C_iv_) extends this approach by incorporating both the volumetric void ratio and the binder content into a unified parameter capable of capturing the combined effects of packing density and bonding.

Accordingly, the η/C_iv_ index has been successfully applied to describe the strength and stiffness of cemented soils, and its use can be extended to alternative binders such as biopolymers. This suggests that, despite the differences in bonding mechanisms between cementitious and polymeric systems, a consistent relationship between mechanical behavior and the η/C_iv_ index can be established for soil–binder mixtures.

### 3.3. Normalization of Equations for the Strength Between Sand–GG and Sand–Type III Portland Cement Mixtures III

Normalization seeks to formulate predictive models capable of reducing the inherent dispersion of individual samples in order to identify a global and unique behavioral trend. In this context, the objective is to establish an appropriate equation to estimate the unconfined compressive strength $q_{u}$ as a function of the normalized index $\eta /{B_{iv}}^{a}$, so that the results can be described by a single potential relationship valid for the different materials analyzed under the various evaluated conditions.

**Figure 10:**
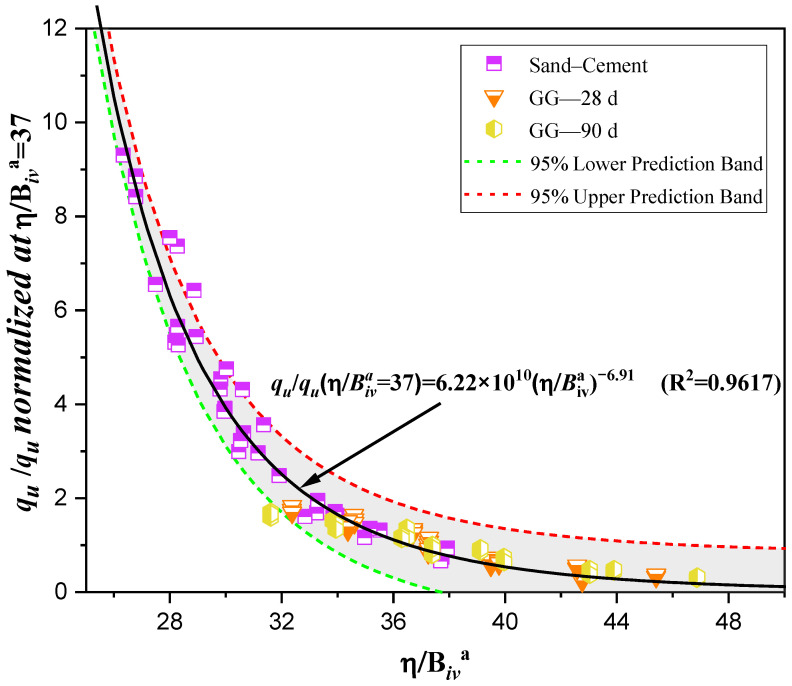
Normalization using the porosity/binder index for UCS $\left(q_{u}\right)$ data.

In previous studies, Baldovino et al. [44] applied normalization to a soil mixed with different percentages of xanthan gum and polypropylene fibers for clay improvement. In that case, to establish a normalized equation, the first step consisted of determining the normalized strengths using a specific value of $\eta /{B_{iv}}^{a}=\nabla$, For the present study, the normalization range of ∇ was established between 25 and 45, and a value of ∇=37 was selected (see Figure 10).

The normalized strengths $q_{u-n}$ were 110.19 kPa for the sand–cement mixture, 190.19 kPa for GG-28d, and 284.91 kPa for GG-90d. After calculating the normalized unconfined compressive strength values, each sample was divided by its corresponding normalized value. Figure 10 together with Equation (6) represents the best fit based on the selected parameter, with a normalization coefficient of 0.96, indicating a unique trend encompassing all experimental data:

(6) $$\frac{q_{u}}{q_{u-n}\left(\eta /{\left(B_{iv}\right)}^{a}=37\right)}=6.22\times 10^{10}{\left(\eta /{\left(B_{iv}\right)}^{a}\right)}^{6.91}\;\left(R^{2}=0.9617\right)$$

The degree of cementation is not solely a function of the amount of cementing agent. Other factors such as initial density, particle size distribution, gradation, texture, and mineralogy also play a significant role in this process. The shear strength of soil–cement mixtures depends on both the degree of cementation and the initial mean effective stress. The mobilization of shear strength in cemented specimens subjected to triaxial loading can be interpreted in terms of the relative contributions of frictional resistance and cementation bonding. When the degree of cementation is high relative to the initial mean effective stress, the contribution of cementation dominates, and the resulting strength approaches the unconfined compressive strength (q_u_). Conversely, when the initial mean effective stress is high relative to the degree of cementation, the frictional component becomes dominant, and the strength approaches that of the uncemented soil.

### 3.4. Statistical Analysis of the Influence of GG Content, Curing, Dry Unit Weight on the UCS of a Sand

Table 3 presents the results of the analysis of variance for the GG-soil mixtures, showing that the variability in UCS is explained by the factors and their interactions, demonstrating that the model is reliable. The individual factors are significant, while the interaction between molding density and GG content is not (*p* = 0.112), indicating that the effect of curing time on strength does not significantly depend on density alone.

### 3.5. Microstructure and Microanalysis of Soil Mixtures

#### 3.5.1. Microstructure and Microanalysis of Sand–GG Mixtures

The experimental SEM–EDS results were obtained from two selected specimens. The first specimen contained 1% GG and was prepared at maximum dry density, while the second contained 0.5% GG and was also prepared at maximum dry density. In both cases, a curing period of 90 days was applied.

**Figure 11:**
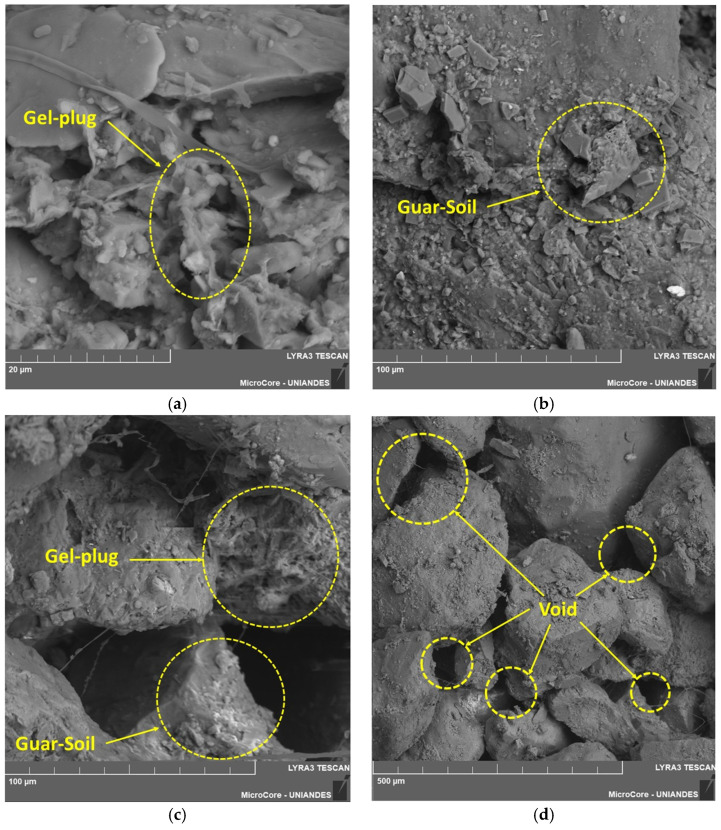
Microscopy of the specimen stabilized with 1% GG, at maximum dry density and 90 days of curing: (**a**) 20 µm scale, (**b**) 100 µm scale, (**c**) 100 µm scale, and (**d**) 500 µm scale.

Figure 11 presents the results for the sand–GG mixture with 1% GG, prepared at maximum dry density and cured for 90 days. Figure 11a shows the formation of hydrogels, while Figure 11b,c illustrate the interaction between the soil particles and the GG, allowing observation of the interface formed because of the homogenization and curing of GG. Figure 11d presents the voids within the newly formed soil–GG matrix, where a noticeable reduction in pore spaces can be observed, resulting in a denser and less porous mixture.

**Figure 12:**
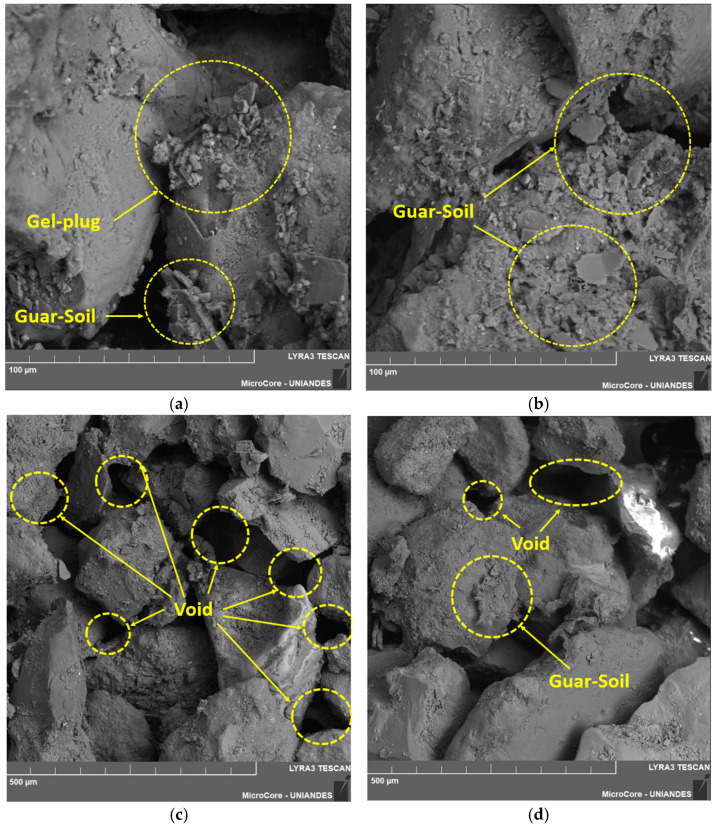
Microscopy of the specimen stabilized with 0.5% GG, at maximum dry density and 90 days of curing: (**a**) 100 µm scale, (**b**) 100 µm scale, (**c**) 500 µm scale, and (**d**) 500 µm scale.

On the other hand, Figure 12a also shows the formation of hydrogels and the new soil composition resulting from mixing the soil with 0.5% GG. In Figure 12b, the remaining voids after the application of GG are observed; in this case, they are larger than those observed in the mixture with 1% GG, which is an expected result due to the reduction in the GG content, as the biopolymer is distributed within the naturally existing soil voids. Figure 12c,d present the interface between the sand particles and GG. Although the GG content is lower than that shown in Figure 11, a clear interaction between the two components is still observed.

**Figure 13:**
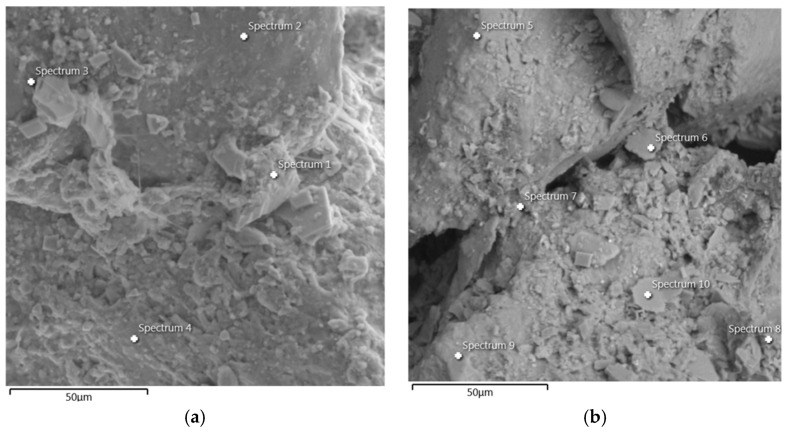
Energy-Dispersive X-ray Spectroscopy (EDS): (**a**) specimen stabilized with 1% GG and (**b**) specimen stabilized with 0.5% GG.

Energy-Dispersive X-ray Spectroscopy (EDS) was used to determine the chemical composition of the two sand–GG samples tested. In this analysis, four spectra were collected for the sand stabilized with 1% GG, while six spectra were obtained for the sample containing 0.5% GG. The locations of these spectra are shown in Figure 13a and Figure 13b, respectively. The results of the elemental percentages and the different chemical elements identified across all ten spectra are presented in Table 4.

The SEM–EDS areas were selected to observe the main features controlling the behavior of the sand–GG matrix, including particle contacts, and zones where the biopolymer could act as a coating or bridge between grains. Several points were analyzed in each sample to avoid relying on a single local feature and to obtain a more representative description of the treated material.

Although SEM images reveal the presence of pores, permeability behavior is also controlled by pore connectivity and the formation of GG-induced binding films, which may reduce fluid flow despite visible voids.

Arabani et al. [51], through a microstructural study, revealed that the addition of guar gum improves the interlocking of soil particles by generating a hydrogel matrix. Additionally, Maleki et al. [52] investigated the behavior of biopolymers during the liquefaction process in sands, explaining that biopolymers form a gel-like structure within the void spaces between particles, increasing cohesion and reducing effective porosity. They concluded that this structure enhances interparticle bonding and promotes more efficient force transfer, thereby delaying phenomena such as liquefaction.

In the spectra corresponding to the soil sample stabilized with 1% GG, oxygen was the most abundant and recurrent element, followed by silicon, while calcium, carbon, magnesium, and aluminum were present at lower levels. Similarly, in the six spectra corresponding to the stabilization using 0.5% GG, the same trend was observed for the most abundant elements, namely oxygen and silicon, whereas sodium, magnesium, and calcium were the least represented elements in the analyzed spectra. In the study conducted by Suárez et al. [23], the chemical composition of the Luruaco sand exhibited high percentages of silicon and aluminum, in addition to oxygen, which was identified as the most abundant element. These elements are consistently observed at high proportions in the spectra obtained for both sand–GG mixtures analyzed in the present study.

#### 3.5.2. Microstructure and Microanalysis of Sand—Type III Portland Cement Mixtures

For the same Luruaco sand previously mentioned, Suárez et al. [23] investigated its behavior and geotechnical improvements when stabilized using a traditional binder such as Type III Portland cement. In this case, the SEM–EDS results correspond to a specimen containing 5% cement, prepared at the maximum dry density determined for this soil and cured for a period of 7 days.

The SEM–EDS results reveal a more solid soil matrix with fewer voids, indicating that the addition of cement reduced the natural porosity of the sand. These voids were filled with chemical compounds, evidenced by needle-shaped microcrystals identified as ettringite, which appear in various dimensions and are dispersed throughout the soil–cement mixture. In addition, the presence of calcium silicate hydrate (C–S–H) was observed, contributing to the confinement and densification within the porous soil matrix [23].

Energy-Dispersive X-ray Spectroscopy (EDS) was used to determine the chemical composition of the sand–cement specimen with a cement content of 5%. In this analysis, two spectra were collected for the sand–cement sample. The elemental percentages and the different chemical elements identified in both spectra are presented in Table 5.

In spectrum 1, the predominant elements were oxygen (42.27%), carbon (20.44%), and calcium (20.07%), followed by silicon (10.50%). This elemental composition is associated with cementitious products such as calcium silicate hydrate (C–S–H) and possible ettringite phases. In spectrum 16, oxygen remained the most predominant element, while carbon continued to appear at a relatively high percentage, and calcium showed a slight decrease compared to spectrum 15, reflecting variations in the distribution of hydration products.

#### 3.5.3. Microstructural and Microanalytical Comparison Between Sand–GG and Sand—Type III Portland Cement Mixtures III

The use of GG as a biopolymer agent for soil stabilization represents a favorable alternative due to its natural availability and sustainability, a condition that is not replicated in the case of Portland cement. However, it is in terms of geotechnical improvements where the suitability of using one binder over the other becomes evident. Through comprehensive experimental testing aimed at comparing both binders and the geotechnical advantages or benefits they provide to the soil, the comparative results of these tests are presented.

For the sample treated with GG, as shown in Figure 11 and Figure 12, an increase in GG content led to a reduction in voids within the mixture. A similar behavior was reported by Kumar et al. [53], who found that GG accumulated within soil voids, coating soil particles and promoting the formation of hydrogels. These hydrogels influenced the strength and density results of the soil–GG matrix. Additionally, the strength of these bonds generated through hydrogel formation increases over time, depending on gel hydration and thickness development [45].

**Figure 14:**
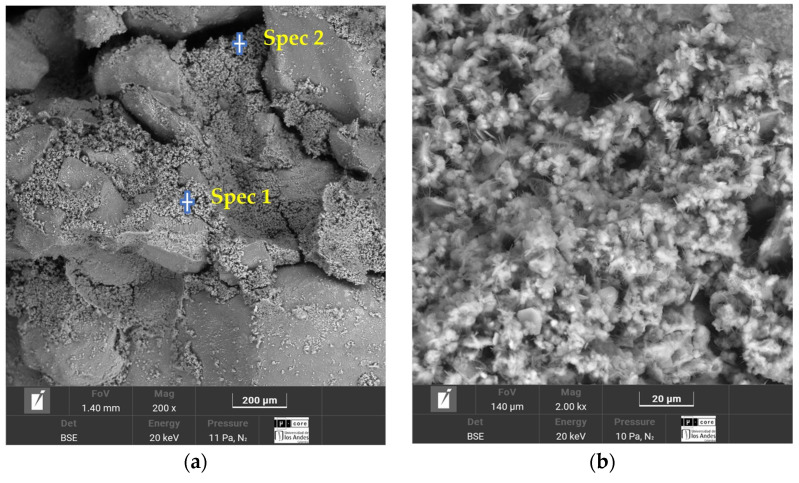
Microstructural analysis of Luruaco sand with 7 days of curing and a cement content of 5%: (**a**) soil-cement matrix and (**b**) ettringite and C–S–H formation [23].

In the case of Type III Portland cement, the results revealed a highly dense matrix in which excessive voids were not observed (Figure 14). Furthermore, the cementitious matrix formed by hydration products such as calcium silicate hydrate (C–S–H) and ettringite provides greater contributions to cohesion and interaction among soil particles. Another factor to consider is that the specimen corresponding to the SEM–EDS results does not represent the maximum cement content used in the study (9%), but rather the third-highest dosage, which was 5%.

Based on the microstructural analysis, the soil–cement mixture exhibited a greater reduction in voids, which translated into improved strength and stiffness of the resulting mixture. However, the cement dosage required to achieve these improvements was considerably higher than that used to stabilize the same soil with GG. From the chemical microanalysis perspective, the elements most favorable for cementation and durability enhancement are calcium (Ca), silicon (Si), aluminum (Al), iron (Fe), and sulfur (S). Calcium showed more consistent results in the cement-stabilized sample, whereas silicon, which is essential for C–S–H formation, was observed in high proportions in the GG–stabilized samples in terms of cementation potential. Considering durability, iron contributes to improved chemical resistance and long-term stability, with GG providing higher iron content compared to the cement-stabilized sample.

Regarding ettringite formation, aluminum and sulfur are the key elements involved in its development. GG-stabilized samples exhibited higher aluminum content, while sulfur was present only in the cement-stabilized sample. However, SEM observations confirmed that ettringite formation was not detected in the GG-treated samples.

Overall, the use of cement resulted in a typical C–S–H-based matrix with ettringite formation, but with lower elemental diversity and limited presence of elements such as iron and aluminum. In this case, cement stabilization provides strong initial cementation due to high calcium contents and the formation of ettringite and C–S–H, which are ideal conditions for short-term mechanical strength development. Conversely, the use of GG exhibits greater elemental richness, particularly in silicon, aluminum, and iron, resulting in a dense and durable matrix associated with hydrogel formation and stable long-term chemical bonding.

The structure of soil–cement initially compresses under loading, as expected; however, with increasing deformation, it exhibits dilation, similar to dense granular materials. This response reflects the dual nature of resistance in cemented sands, where both bonding and friction contribute to the overall mechanical behavior. The porosity/binder index (η/B_iv_) provides a suitable framework to capture this transition. At lower η/B_iv_ values (denser and more bonded systems), cementation dominates the response, leading to higher stiffness and peak strength. As density increases, however, the contribution of cementation becomes less significant relative to frictional resistance, since a greater number of particle contacts enhances intergranular friction. Additionally, the role of cementation is more pronounced at low confining pressures, where bonding contributes to an expansion of the yield surface. At higher confining stresses, bond breakage and particle rearrangement reduce the effectiveness of cementation, and the behavior converges toward that of uncemented granular soils. This highlights the capability of the η/B_iv_ index to describe not only strength variations but also the evolving balance between bonding and frictional mechanisms.

The specimens were cured in sealed plastic bags to reduce moisture loss and maintain controlled laboratory conditions during the curing period. This procedure was adopted to isolate the effect of GG on the mechanical and microstructural response of the treated sand. However, it should be noted that these curing conditions do not fully reproduce field conditions, where treated soils may be exposed to moisture fluctuations, drainage processes, and repeated wetting–drying cycles. Since GG develops its bonding capacity mainly through hydrogel formation and polymeric bridges between soil particles, variations in moisture content may affect the stability of these bonds and reduce strength over time. Therefore, the results reported in this study should be interpreted as representative of controlled curing conditions. Further studies involving wetting–drying cycles and field-scale exposure are recommended to evaluate the long-term durability of GG-treated soils under real environmental conditions.

## 4. Discussion

The results show that GG improved the UCS of the poorly graded sand, but its behavior was clearly different from that observed in Type III Portland cement mixtures. In the GG-treated specimens, the strength increase from 28 to 90 days suggests a gradual stabilization process controlled by hydrogel development, particle coating, and the formation of polymer bridges. This agrees with previous studies, which show that GG improved soil strength mainly by creating hydrated gel bonds between particles and increasing apparent cohesion [45,52,54].

A similar response has been reported for aeolian and desert sands treated with GG, although the optimum dosage and curing period vary depending on soil gradation, moisture condition, and target application [54,55]. Therefore, the performance obtained in this study should be interpreted as part of a broader trend: GG can improve granular soils, but its efficiency depends strongly on how well the polymer is distributed within the voids and particle contacts.

The comparison with cement requires caution. Cement produced a higher early UCS because its hydration reactions generate rigid products such as C–S–H and ettringite in a short time. GG, instead, needs longer curing to develop a measurable bonding network. Thus, the results do not indicate that GG is equivalent to cement at the same curing age, but rather that it can reach strength levels comparable to low cement contents when longer curing and controlled moisture conditions are available.

The η/B_iv_ index helped describe the UCS response of both systems, even though their bonding mechanisms are different. In cemented sand, lower η/B_iv_ values are related to denser packing and greater formation of cementitious products. In GG-treated sand, the same trend reflects the combined effect of lower porosity and higher availability of hydrated polymer to form bridges between grains. This supports the use of the index as a useful laboratory tool, since strength is not governed only by binder content but also by density, porosity, particle arrangement, and bonding continuity [23,24,43,44].

Even so, the proposed equations should not be taken as direct field-design expressions. They are valid within the tested range of density, GG content, curing time, and soil type.

The SEM observations are consistent with the mechanical results. The GG-treated specimens did not develop a continuous mineral matrix like cemented sand, but showed coated grains, hydrogel bridges, and remaining pores. This type of structure is expected in biopolymer-treated granular soils. Visible pores do not necessarily mean poor bonding, since GG may still improve particle contact and reduce pore connectivity through localized gel formation [52,56,57].

This is important when interpreting the possible permeability behavior. SEM images provide local evidence of pores, but they do not define the real flow paths of the material. Permeability depends on pore size, tortuosity, and connectivity. Ai et al. [54] reported a strong reduction in permeability in GG-treated aeolian sand, even though changes in the pore structure were still visible. Similar mechanisms were described in dispersive saline soil treated with GG, where the hydrogel contributed to void filling and stronger particle interaction [58]. For this reason, direct permeability tests are still needed in the present sand.

The main limitation of GG stabilization is its sensitivity to moisture conditions. Since its bonding capacity depends on hydration and hydrogel stability, wetting–drying cycles may damage part of the soil–polymer interface. Previous studies have shown that biopolymer-treated soils can lose strength after cyclic wetting and drying due to swelling, shrinkage, and partial bond disruption [47,59]. Other works have also reported microstructural deterioration under dry–wet or freeze–thaw exposure, depending on polymer type, dosage, curing time, and soil texture [60,61].

From a practical point of view, GG should not be presented as a direct replacement for cement in applications requiring rapid and high early strength. Its contribution is more suitable for cases where moderate strength gain, low binder dosage, and environmental compatibility are relevant. Similar conclusions have been reported for GG-stabilized loess and expansive subgrades, where the biopolymer improved cohesion, erosion resistance, CBR, and water retention, but remained dependent on dosage and environmental exposure [62,63]. Therefore, GG can be considered a promising alternative for sandy soil stabilization, provided that future studies address permeability, durability, and field-scale performance.

## 5. Conclusions and Recommendations

This study compared the mechanical performance, predictive modeling capability, and microstructural characteristics of stabilizing sand with GG and Portland cement under controlled curing conditions. The main conclusions derived from this study are presented below:

- GG showed its best performance at higher dosages and longer curing periods (90 days), reaching strengths of up to 470 kPa, comparable to those obtained with cement at 3% and 5% after 7 days of curing. However, cement still achieved strengths above 700 kPa due to the formation of a rigid cementitious matrix. GG can therefore be considered a sustainable alternative for sand stabilization when longer curing times are acceptable and only moderate mechanical performance is required.
- The porosity/binder index successfully predicted the unconfined compressive strength of sand stabilized with both GG and cement, independent of curing time. The models showed high reliability, with R^2^ values of 0.9231 and 0.9196 for GG (28 and 90 days) and 0.9525 for cement. The internal exponent a was determined to be 0.29 for GG and 0.25 for cement, confirming the validity of the index across different types of binders in granular systems.
- The normalization of an equation describing the unconfined compressive strength for all specimens yielded a coefficient of determination $\left(R^{2}\right)$ of 0.9617. Considering that the two binders are of different types—one being a conventional material and the other a recently studied natural biopolymer—the correlation obtained between both allows their behavior to be unified by a single equation.
- The microstructure of the soil–cement mixture exhibited a denser matrix than that of the soil–GG mixture. This result was expected, given that cement stabilization involves solid mineral precipitation, which contributes to an increase in the real solid volume. In contrast, GG stabilization primarily involves pore occupation through hydrogel formation, and a portion of the mixture volume is composed of retained water.

Although the results demonstrate promising short- to medium-term performance (up to 90 days), the long-term durability of GG-treated soils, particularly beyond 180 days, requires further investigation due to potential biodegradation effects. Additionally, for future studies, it is recommended to conduct permeability tests to directly evaluate the behavior of the pores after stabilizing the soil with guar gum.

## Figures and Tables

**Table 1:** Geotechnical properties of the soil sample.

Property of Soil	Standard/Reference	Unit	Value
Maximum γ_d_	[27]	kN/m^3^	1.78
Minimum γ_d_	[28]	kN/m^3^	1.52
Specific Gravity, Gs	[26]	-	2.73
Gravel (4.75–76.2 mm)	[25]	%	10.8
Coarse Sand (2.00–4.75 mm)	[25]	%	20.1
Medium Sand (0.425–2.0 mm)	[25]	%	42.3
Fine Sand (0.075–0.425 mm)	[25]	%	26.6
Silt (0.002–0.075 mm)	[25]	%	0.2
Mean Diameter (*d*_50_)	[25]	mm	0.85
Effective Diameter (*d*_10_)	[25]	mm	0.20
Uniformity Coefficient *C*_u_	[25]	-	5.9
Coefficient of Curvature *C*_c_	[25]	-	0.7
USCS Classification	[25]	-	SP

**Table 2:** Design of mixed dosages for compacted soil–GG and soil-cement mixtures.

Mix	Weight (%)			Curing Times (d)	Molding γ_d_ (g/cm^3^)	Number of Specimens
	Soil	GG	Cement			
Soil–GG	100	0.25	-	28, 90	1.71, 1.65, 1,58	18
	100	0.50	-	28, 90	1.71, 1.65, 1,58	18
	100	0.75	-	28, 90	1.71, 1.65, 1,58	18
	100	1.00		28, 90	1.71, 1.65, 1,58	18
Soil–Cement	100	-	3	7	1.71, 1.65, 1,58	9
	100	-	5	7	1.71, 1.65, 1,58	9
	100	-	7	7	1.71, 1.65, 1,58	9
	100	-	9	7	1.71, 1.65, 1,58	9

**Table 3:** ANOVA table for UCS results of GG mixtures.

Source	Sum of Squares	Degrees of Freedom	Mean Squares	Z	*p*-Value	Significance (*p*-Value < 0.05)
Corrected Model	963,438.596	22	43,792.663	151.816	<0.001	yes
Intercept	2,722,468.993	1	2,722,468.993	9437.955	<0.001	yes
Density	160,336.386	2	80,168.193	277.918	<0.001	yes
GG	609,176.159	3	203,058.720	703.942	<0.001	yes
Curing time	129,815.542	1	129,815.542	450.030	<0.001	yes
Density * GG	7065.981	6	1177.663	4.083	0.003	yes
Density * Curing time	1332.513	2	666.257	2.310	0.112	no
GG * Curing time	28,848.432	3	9616.144	33.336	<0.001	yes
Density * GG * Curing time	3647.970	5	729.594	2.529	0.043	yes
Error	12,115.305	42	288.460			
Total	4,364,288.480	65				
Corrected Total	975,553.901	64				

* R^2^ = 0.988 (R^2^ corrected = 0.981).

**Table 4:** Elements and percentages identified in the ten spectra.

Element	Wt% (Weight Percent)									
	Spec 1	Spec 2	Spec 3	Spec 4	Spec 5	Spec 6	Spec 7	Spec 8	Spec 9	Spec 10
C	7.93	21.53	15.19	15.27	-	-	7.98	7.79	11.98	-
O	50.76	33.64	49.45	35.36	58.12	40.80	35.84	27.35	57.31	61.11
Mg	8.72	-	0.45	-	-	4.62	1.25	0.68	0.33	-
Al	6.42	-	1.18	0.24	-	11.59	4.73	11.72	0.70	15.15
Si	16.53	44.84	30.81	17.22	41.88	18.77	13.81	30.15	26.73	19.86
Ca	4.60	-	-	22.07	-	-	1.34	6.54	-	-
Fe	5.05	-	2.91	2.13	-	20.97	32.39	12.8	2.94	-
Na	-	-	-	0.36	-	-	1.42	1.96	-	-
K	-	-	-	7.34	-	3.24	1.24	1.01	-	3.88

**Table 5:** Elements and percentages identified in the two spectra.

Element	Wt% (Weight Percent)	
	Spec 1	Spec 2
Aluminum	2.18	2.90
Calcium	20.07	17.41
Carbon	20.44	19.10
Iron	2.50	5.31
Magnesium	0.78	0.38
Oxygen	42.27	41.93
Potassium	0.46	0.35
Silicon	10.50	12.41
Sodium	-	0.17
Sulphur	0.80	0.03

## Data Availability

Data are contained within the article.
